# Intraobserver and Interobserver Agreement between Six Radiologists Describing mpMRI Features of Prostate Cancer Using a PI-RADS 2.1 Structured Reporting Scheme

**DOI:** 10.3390/life13020580

**Published:** 2023-02-19

**Authors:** Rafał Jóźwiak, Piotr Sobecki, Tomasz Lorenc

**Affiliations:** 1 Applied Artificial Intelligence Laboratory, National Information Processing Institute, 00-608 Warsaw, Poland; 2 Faculty of Mathematics and Information Science, Warsaw University of Technology, 00-661 Warsaw, Poland; 3 Department of Clinical Radiology, Medical University of Warsaw, 02-091 Warszawa, Poland

**Keywords:** prostate cancer, structured reporting, PI-RADS

## Abstract

Clinical practice has revealed ambiguities in PI-RADS v2.1 scoring, but a limited number of studies are available that validate the interreader and intrareader reproducibility of the mpMRI PI-RADS lexicon. We decomposed the PI-RADS rules into a set of common data elements to evaluate the inter- and intraobserver agreement in assessing the individual features included in the PI-RADS lexicon. Six radiologists (three highly experienced, three less experienced) in two sessions independently read thirty-two lesions in the peripheral and transition zone using the structured reporting tool, blinded to clinical MRI indication. The highest agreement between radiologists was observed for the abnormality detection, the evaluation of the type of signal intensity, and the characteristic of benign prostatic hyperplasia. Moderate agreement was reported for dynamic contrast-enhanced images. This resulted in a decrease in abnormality detection (PA = 76.5%) and enhancement indication (PA = 77.3%). The lowest agreement was observed for highly subjective features: shape, signal intensity level, and type of lesion margins. The results indicate the limitations of the PI-RADS v2.1 lexicon in relation to interreader and intrareader reproducibility. We have demonstrated that it is possible to develop structured reporting systems standardized according to the PI-RADS lexicon.

## 1. Introduction

Due to the increasing incidence rate in the last decade, prostate cancer has become a growing public health concern worldwide [1]. Early detection of prostate cancer can greatly improve patient prognosis. Recent advantages in MRI technology that allows both anatomical and functional imaging to be performed simultaneously, and multiparametric magnetic resonance imaging (mpMRI) has improved our ability to detect and characterize prostate tumors [2]. As an important adjunctive tool to clinical assessment, magnetic resonance imaging has shown great potential to diagnose prostate masses, especially in elevated PSA cases, allowing the identification of masses that are occult on ultrasound and systemic biopsies [3]. According to patient management guidelines, non-invasive diagnostics, such as mpMRI, play an important role in the referral of patients to active surveillance, watchful waiting [4,5], and radical prostatectomy [6].

To create a global standard in the acquisition, interpretation, and reporting of prostate mpMRI examinations, a standardized prostate MRI assessment prostate imaging reporting and data system called PI-RADS was released [7]. To improve the detection, localization, and risk stratification in patients with treatment-naïve prostate glands, these guidelines were updated to PI-RADS version 2 [8]. Ambiguities in the scoring and limitations in relation to reproducibility were problematic disadvantages of this system; therefore, guidelines were updated in version PI-RADS v2.1 [9]. The system is based on the calculation of points for the evaluation of each focal lesion with different sequences, namely T2-weighted imaging (T2WI), diffusion-weighted imaging (DWI), and dynamic contrast-enhanced (DCE), and these scores are used to calculate an overall assessment category. However, low specificity and high interobserver variability remain problematic disadvantages of MRI, especially for non-dedicated or less experienced radiologists who received only short-term training in prostate MRI [10]. Although the American College of Radiology proposed the prostate imaging report and data system (PI-RADS) lexicon, most radiologists are still inclined to have relatively poor diagnostic performance when assessing prostate lesions. Numerous studies have ratified the value of PI-RADS v2 [11,12,13], but a limited number of studies on PI-RADS v2.1 are available.

The structure of a radiological report and its organization should ensure that all relevant areas are addressed. Basically, the term ‘structured report’ refers to the organization of the text itself into a preformatted structured document, with separate sections for clinical information, examination protocol, radiological findings, and conclusions. The radiology community has recognized the need to create standard terminology to improve the clarity of reports and to reduce radiologist variation [14,15]. According to Nobel et al., structured reporting should be considered a set of IT-based tools to implement a structured form of reporting, to the ultimate benefit of both radiological and general clinical practice [16]. In the context of the development of modern structured reporting, the problem of interoperability is especially pertinent. The concept of common data elements (CDEs) in radiology improves the process of data organization and management in structured reporting. A common data element is a unit of information used in a shared and predefined manner [17]. Generally, the term ‘CDE’ pertains to standardized key terms in a given application area, comparable to an attribute; CDEs can act as keys, which can then map to associated values, e.g., shape–oval.

The objective of our study was to evaluate the inter- and intraobserver agreement among radiologists with different levels of experience using a structured report scheme, in which CDEs were defined on the basis of a standardized PI-RADS v2.1 lexicon and assessment categories.

## 2. Materials and Methods

### 2.1. Dataset

The study involved a selected group of cases from a publicly available database of mpMRI data for prostate lesion classification, which was originally created for the PROSTATEx Challenge (SPIE-AAPM-NCI Prostate MR classification Challenge) held in conjunction with the 2017 SPIE Medical Imaging Symposium [18]. All cases underwent a histopathological evaluation. We proposed an experiment group diversified according to the lesion and its clinical significance. Among all, 14 lesions were located in the peripheral zone (PZ) (7 clinically significant and 7 not clinically significant), 11 lesions were located in the transitional zone (TZ) (5 clinically significant and 6 not clinically significant), and 7 lesions were located in the anterior fibromuscular stroma (AFMS) (4 clinically significant and 3 not clinically significant). All studies in the ProstateX database included T2-weighted (T2W), DCE, and diffusion-weighted (DW) images. Images were acquired on two different types of Siemens 3T MR scanners, the MAGNETOM Trio and Skyra. The T2-weighted images were acquired using a turbo spin echo sequence and had a resolution of around 0.5 mm in the plane and a slice thickness of 3.6 mm. The DCE time series were acquired using a 3D turbo flash gradient echo sequence with a resolution of around 1.5 mm in the plane, a slice thickness of 4 mm, and a temporal resolution of 3.5 s. Finally, the DWI series were acquired with a single-shot echo planar imaging sequence with a resolution of 2 mm in the plane and 3.6 mm slice thickness and with diffusion-encoding gradients in three directions. Three b values were acquired (50, 400, and 800), and subsequently, the ADC map was calculated by the scanner software. All images were acquired without an endorectal coil.

### 2.2. Structured Report Scheme

**Figure 1:**
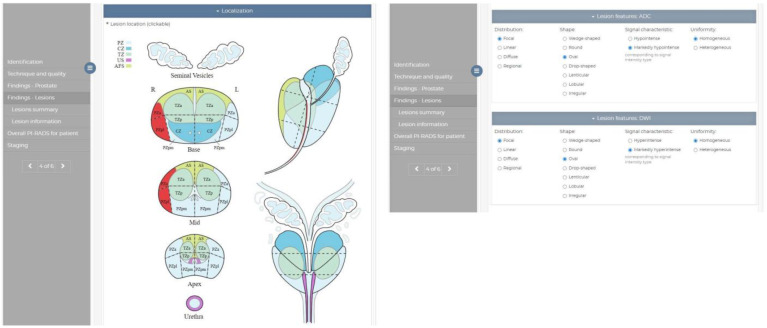
Example views of the created SR from, implemented as a module in the eRADS system. The left part presents an interactive picture supporting selection of lesion localization, while on the right there is an interactive panel for evaluation of lesion individual features (in that case on ADC/DWI sequences).

We propose a standardized structured report form for reporting mpMRI examinations in prostate cancer. The proposed set of CDE elements was defined by the decomposition of the PI-RADS v2.1 narrative guidelines into single elements that appear in the PI-RADS lexicon (relating to various categories, including abnormality, shape, margins, signal characteristics, etc.), as well as elements that exceed the standard and refer to clinically significant features or morphometric lesion features. Most of the proposed CDE can be identified in the RadLex lexicon; however, in a few cases, it was also necessary to introduce additional variables that were not included in the RadLex lexicon. Based on the insights of the radiologists, two additional CDEs were incorporated to simplify the rule sets. According to experts, the assessment of particular shape and margin type features in mpMRI images is highly subjective. Instead, categorization was suggested, mapping the particular values into more general feature types to simplify the defined rules. As a result, eight shapes described as part of the PI-RADS lexicon (round, oval, lenticular, lobulated, tear-shaped, wedge-shaped, linear, and irregular) were simplified to three shape types (linear, round, and irregular). At the same time, different types of margins were grouped into non-circumscribed (indistinct, obscured, spiculated, encapsulated, and erased charcoal sign) and circumscribed (encapsulated, partly-encapsulated, and well-defined). Configuration of PI-RADS v2.1-inspired CDEs is presented in Table 1. The structured report form was implemented as an interactive electronic form, composed of a dedicated collection of radio buttons or checkboxes, with labels related to the proposed CDE, identified as specific to prostate cancer. The elaborated form was published as a module on the dedicated platform for radiological structured reporting, eRADS (https://opi.org.pl/en/systems/erads/description/ (accessed on 30 December 2022)). Figure 1 presents selected parts of the created SR form.

### 2.3. Radiological Assessment

The experiment was conducted with the participation of highly experienced and less experienced radiologists. These experts were not involved in the methodology development process. The study was carried out in a group of radiology specialists who used PI-RADS standards during the diagnostic practice:

- Three specialists with diagnostic experience of one to five years;
- Three specialists with more than ten years of diagnostic experience and at least five years of experience using the PI-RADS standard (since the first version of the standard).

The specialists were instructed not to contact each other during the study to discuss the cases they had assessed. The study involved two sessions that required the complete assessment of thirty-two selected lesions using the proposed structured report form. In the first phase, the radiologists assessed all lesions by specifying the imaging features (the values of the identified CDEs) and assigned the manual PI-RADS categories. The second phase was conducted two weeks after the first to eliminate the memory effect and to allow intra-reader analysis. The time spent in interaction with the computer-assisted reporting form during each assessment of the mpMRI examination was automatically measured. After completing the examinations, we collected the opinions of the radiologist participating in the study.

We collected opinions of the diagnosticians participating in the test, who pointed to a number of usability advantages, including verification of inference through suggestions for compliance with diagnostic guidelines, simplicity of report creation (minimizing the use of the keyboard in favor of the mouse when completing the form), and clarity and uniformity of the resulting textual reports.

### 2.4. Statistical Analysis

We statistically analyzed the results of interrater agreement of the concept of common data elements based on the PI-RADS v2.1 lexicon. We present the percent concordance (PA) and the first-order agreement coefficient (AC1) obtained using Gwet’s method for the CDEs [19]. Additionally, the intrarater agreement was estimated using the same measures by comparing the assessments between the two study sessions performed on the retrospective data. Statistical significance levels were set at 5% and the interpretation of the agreement levels was defined as excellent for AC1 values (≥0.81), good (0.61–0.80), moderate (0.41–0.60), fair (0.21–0.40), and poor (≤20) [20]. We used a Wilcoxon signed rank test to compare the means of the assessed features. AUC, recall, and precision were used as measures of the performance of the diagnostic methods. Data cleaning, restructuration, and visualization were performed in Python (v3.7.12) using Pandas (v1.3.5) and Plotly (v5.5.0) packages. Statistical analysis was performed using the package R (v4.1.2) and irrCAC (v1.0) package [21]. All scripts were written in the Google Collaboratory tool using the dedicated notebooks.

## 3. Results

In this section, we present the results of the retrospective study conducted on thirty-two prostate lesions drawn from the ProstateX training dataset. These lesions were pre-selected for evaluation by six radiologists.

### 3.1. Interrater Agreement

Based on the results obtained from the two stages of the retrospective study, the mean interrater percentage agreement and AC1 values with 95% confidence intervals are presented in Table 2 for estimated values of PI-RADS v2.1 CDEs. Overall, the table presents the mean of fifteen pairs of radiologists’ evaluations that were compared to estimate their concordance.

The highest agreement between the radiologists was observed for abnormality detection, assessment of signal intensity type, and benign prostatic hyperplasia (BPH) feature CDEs. Results were expected for the first two characteristics since radiologists received reference images of abnormalities in all modalities as a guide. The type of signal intensity is strongly associated with the occurrence of lesions in these modalities; hypointensity indicated the abnormalities for the T2W and ADC images and hyperintensity for the DWI images. Given a study design that assesses the potentially clinically significant lesions selected, these characteristics demonstrated high agreement between the raters; this, however, was not observed for the DCE images, for which abnormalities were not observed in all cases analyzed. This resulted in a decreased agreement in the detection of abnormalities (PA = 76.5%) and enhancement indication (PA = 77.3%), suggesting that not all abnormalities are evident in all mpMRI sequences and that the evaluation of signal enhancement on DCE is subjective. The lowest agreement was observed for highly subjective features: shape, signal intensity level, and type of lesion margins. The simplification of the shape of the lesion and margin features by grouping the values into types improved the concordance between the raters.

**Figure 2:**
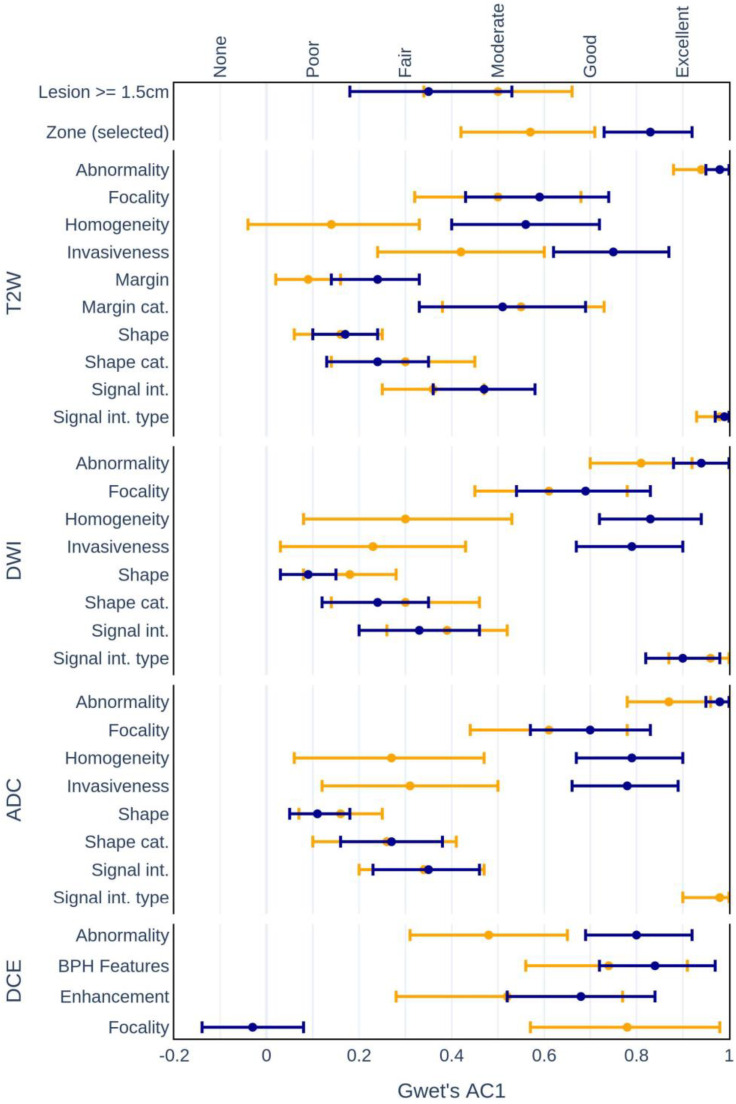
Mean interrater agreement among experienced (blue) and inexperienced (yellow) radiologists.

Analysis of differences in interrater agreement among the experienced and inexperienced raters (within groups) reveals several significant differences in agreement values [Figure 2]. The results present mean comparisons of five pairs of assessments for the experienced and inexperienced groups, each of which was represented by three experts.

The largest difference between the groups was observed in their assessment of focality in DCE images. Agreement among inexperienced raters was not statistically significant (AC1 = −0.03, *p* = 0.37) and was good for the experienced raters (AC1 = 0.78, *p* < 0.001). However, this was the only case in which the experienced raters agreed more on the feature assessment. The opposite tendency was observed for:

• Zonal locations of lesions:
– Experienced AC1 = 0.57, *p* < 0.001 vs. Inexperienced AC1 = 0.83, *p* < 0.001.
• Homogeneity on:
– T2W (AC1 = 0.14, *p* = 0.13 vs. AC1 = 0.56, *p* < 0.001);
– DWI (AC1 = 0.30, *p* < 0.01 vs. AC1 = 0.83, *p* < 0.001);
– ADC (AC1 = 0.27, *p* < 0.05 vs. AC1 = 0.79, *p* < 0.001).
• Invasiveness on:
– T2W (AC1 = 0.42, *p* < 0.001 vs. AC1 = 0.75, *p* < 0.001);
– DWI (AC1 = 0.30, *p* < 0.01 vs. AC1 = 0.83, *p* < 0.001);
– ADC (AC1 = 0.27, *p* < 0.05 vs. AC1 = 0.79, *p* < 0.001).
• Abnormality detection on:
– DCE (AC1 = 0.48, *p* < 0.001 vs. AC1 = 0.80, *p* < 0.001).

**Figure 3:**
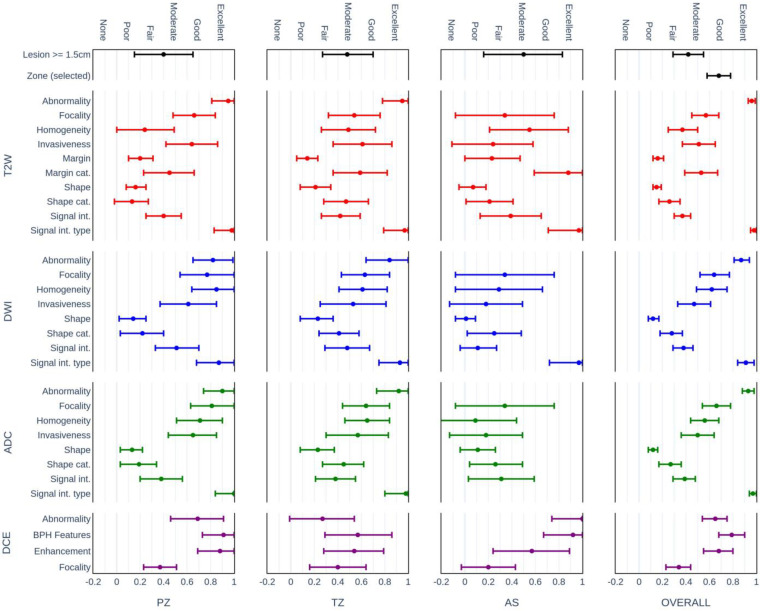
Mean interrater agreement (AC1) of composite PI-RADS CDEs in the PZ, TZ, and AFMS zones and overall results. The colors correspond to the modality sources of the features: T2W (red), DWI (blue), ADC green), and DCE (purple).

Figure 3 presents the concordance analysis of each CDE evaluated on lesions located in the PZ, TZ, and AFMS in comparison to the overall results. The results reveal that the evaluation of AFMS lesion features demonstrated a lower agreement among raters in comparison to PZ and TZ lesions. The overall wider range of confidence intervals can be explained partially by the smaller number of lesions evaluated in the AFMS zone.

Overall, it was observed that the shape and signal intensity features demonstrated the lowest agreement between raters. Analysis of interrater agreement dependent on lesion locations indicates that overall no statistically significant differences were demonstrated in the agreement between PZ (mean PA = 69.7%), TZ (mean PA = 67.3%), and AFMS (mean PA = 65.5%) features. The agreement between the radiologists showed high variability. The highest deviations in agreement between experts were observed for features of the lesions located in the AFMS. This was particularly visible for highly subjective features based on the evaluation of signal intensity, focality, and texture features (homogeneity).

### 3.2. Intrarater Agreement of Defined CDEs

**Figure 4:**
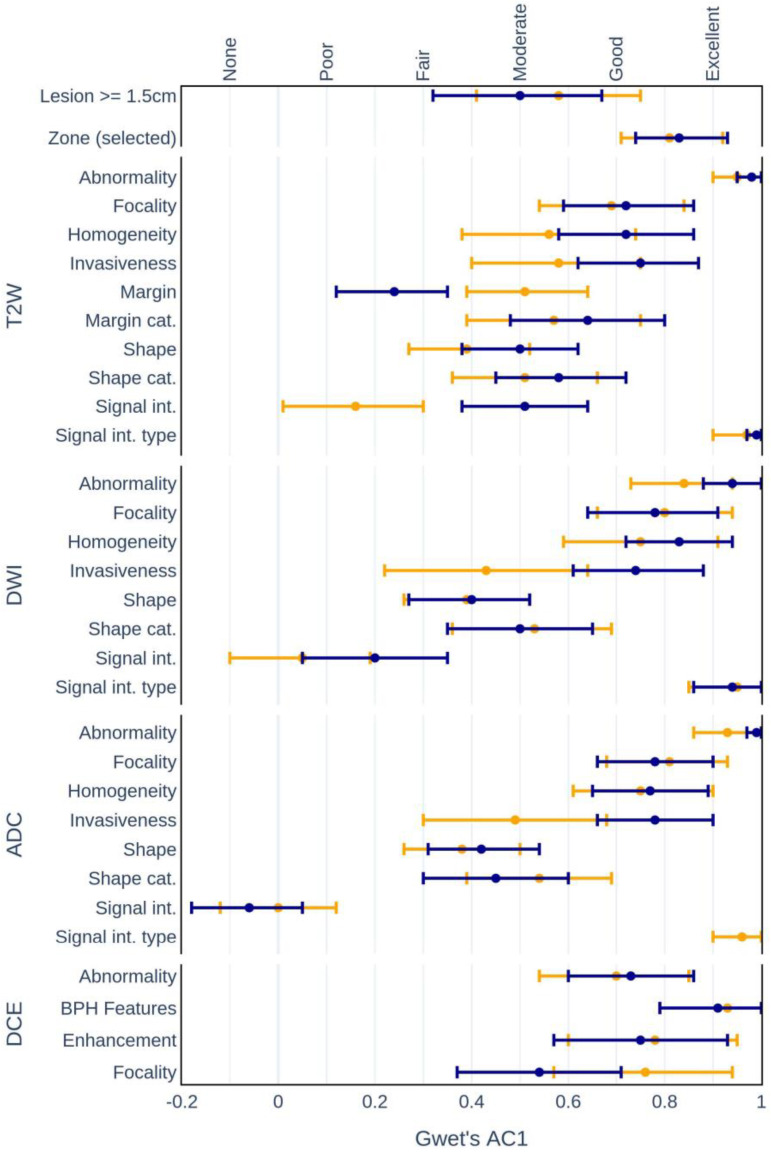
Mean intrarater agreement (AC1) among the experienced (blue) and inexperienced (yellow) raters with 95% confidence intervals.

The analysis of intrarater agreement indicated that most of the feature evaluations displayed moderate or good agreement between the study stages [Figure 4]. The lowest intrarater agreement was observed for the highly subjective low-level features (except homogeneity estimation). For example, the signal intensity evaluation, in which the repeated feature estimation on the ADC images showed no significant agreement in rater estimations between the study sessions. Overall, inexperienced raters displayed higher consistency in their evaluations compared to experienced radiologists, except for the focality assessment of DCE images.

### 3.3. Agreement of the Assessment Categories

Statistical analysis of interrater agreement of the PI-RADS categories for the same evaluated lesions between stages [Table 3] was generally fair to moderate (0.2 < AC1 < 0.6). The highest percentage agreement was observed for the DCE PI-RADS evaluation, but it is crucial to note that this type of evaluation allows only three outcomes: positive, negative, and unavailable (X). The overall PI-RADS scores assigned by experienced radiologists (mean = 4.58, standard deviation = 0.71) to clinically significant lesions were higher (Z = 147, *p* < 0.001) than those assigned by the inexperienced radiologists (mean = 4.09; standard deviation = 1.05).

Table 4 presents the intraobserver agreement of the PI-RADS v2.1 category evaluations according to the T2W, DWI, DCE, and overall algorithms between the study stages. All scoring methods demonstrate similar, moderate statistically significant (*p* < 0.001) intraobserver agreement with respect to AC1 scores.

### 3.4. Diagnostic Accuracy Based on Category Assessment

To investigate the quality of the radiologists’ diagnoses, we used the manually assessed PI-RADS v2.1 categories as a measure of the probability of each lesion’s clinical significance. Diagnostic accuracy was assessed by assuming the EAU guidelines of consideration for patient active treatment, in which PI-RADS >= 3 lesions were considered clinically significant and recommended to be histopathologically evaluated.

The AUC results suggest that despite the lower interrater agreement between the experienced radiologists in both estimated features and PI-RADS category assessment; their diagnoses demonstrated superior performance compared with that of the inexperienced radiologists. This was applied to lesions located in all zones. The evaluations of the experienced radiologists showed higher sensitivity (recall = 0.97 vs. 0.85) and precision (0.61 vs. 0.58). Overall, the diagnostic decisions demonstrated excellent sensitivity (>0.81, mean = 0.91); the precision, however, was moderate (0.5 < precision < 0.67, mean = 0.58). The maximum observed specificity was 0.50 and the lowest was 0.06 (mean = 0.34). No statistically significant differences were observed between the results of the first and second stages in terms of assessment quality.

### 3.5. Radiologists’ Opinions on System

The interviews we conducted with specialists who had interacted with the SR system provided us with information on clinical usability and radiological workout. Radiologists confirmed the potential of the tool in increasing the availability and reliability of diagnostic standards in clinical practice. The tool allowed radiologists to verify the parameters entered in case of discrepancies between the manual and suggested assessments. Both experienced and inexperienced professionals noted the potential of the tool in supporting compliance with diagnostic standards for radiologists in training. Experienced radiologists pointed out that the greatest benefit would provide a solution that reduces the time needed to assess the examination (primarily the time needed to prepare the examination report after visual assessment of the imaging).

## 4. Discussion

Radiologists differ in their assessment of lesions’ qualities, number, and the probability of their clinical significance. Our research has shown that inexperienced radiologists tend to underestimate the PI-RADS assessment scores of clinically significant lesions compared to experienced radiologists. This has also been noticed in the work by Mussi et al., which indicates that moderately experienced raters were more likely than highly experienced to score lesions inconclusively in PI-RADS 3 category rather than indicating their clinical significance (PI-RADS 4 and 5) [20]. These findings suggest that studies on the consistency of PI-RADS evaluation are important, as possible differences in diagnosis contribute to lower recall rates and, thus, the possibility of not identifying clinically significant lesions. Introducing dedicated computational indicators that estimate confidence in cases of inconclusive assessment could improve diagnostic accuracy.

The results show that high-level features that require expert knowledge and subjective interpretation demonstrate decreased agreement between raters. During the interviews, the radiologists established that there was disagreement in their interpretations of the ‘invasiveness’ characteristic. For some, that feature indicates an extraprostatic extension behavior; for others, that definition also incorporates lesions that extended to the surrounding zones/sectors. According to the PI-RADS standard, the latter interpretation is correct when considering the assessment rules. High concordance was observed for other high-level characteristics, including part of the DCE algorithm and evaluation of ‘BPH characteristics’, which indicates that the gland presents features of benign prostate hyperplasia. In general, experienced radiologists showed less agreement than inexperienced ones. This was particularly evident in their evaluations of invasiveness and homogeneity at all stages and focality at the second stage. This contradicts other findings, in which less experienced raters displayed inferior agreement when evaluating MRI features [20]. The unique methodology of our study provided an interobserver and intraobserver analysis of PI-RADS v2.1 category assessments for individual mpMRI sequences. Observed moderate intraobserver agreement for all mpMRI sequences indicates limited repeatability among all features, which may result from the complexity and multimodal nature of the data. Agreement between observers, generally poor for T2W and DWI/ADC and only moderate for DCE, correlates in this case with the number of features evaluated for each of the mpMRI sequences. T2W, which requires the assessment of the largest number of highly subjective low-level features, is characterized by the lowest agreement. At the same time, DCE limited mainly to the high-level assessment of enhancement, is characterized by the highest agreement.

Due to the subjective nature of the mpMRI assessment, interrater agreement varies for particular features. No ‘gold standard’ can be defined by the estimations of a particular radiologist. To construct a reference dataset and ensure high-quality annotations, a committee of experienced diagnosticians would have to participate in rating a substantial dataset of prostate mpMRI. Then, such data could be used to enhance the formal model using radiomics to provide objective measures and confidence levels for the features. Setting a gold standard with the help of an expert panel was beyond our organizational and financial capacity.

Analysis of interrater agreement performed on the results of the retrospective study reveals that although both experienced and inexperienced raters differed in their evaluations of the PI-RADS categories for the preselected lesions, their evaluations demonstrated high recall scores. The results indicate that the method shows low specificity, which means that the diagnosis of mpMRI using the PI-RADS standard can lead to many unnecessary biopsies. Significant differences in the diagnoses of the predictive value of the experienced and inexperienced radiologists can be explained by the low agreement between specialists in assessing high-level features that indicate the clinical significance of a lesion, such as invasiveness. The correct evaluation of these traits requires experience in PCa diagnosis.

We have demonstrated that data collected through interaction with our system during PCa assessment can be used to analyze the characteristics of features that comprise the PI-RADS guidelines. It is possible to identify the descriptors that characterize poor intra- and interrater agreement; these could potentially benefit from redefinition in radiological lexicons or from the integration of automatically quantified image features.

These results are in line with those obtained by Kim et al., who observed poor interreader agreement and a lack of improvement in diagnostic between PI-RADS v2.0 and v2.1 for mpMRI of the transition zone [22]. Moreover, interreader agreement for PI-RADS v2.1 scores had lower (0.26) compared to v2.0 (=0.37). The authors indicated an ongoing need to refine the evaluation of TZ lesions. On the other hand, Urase et al. observed better agreement among all readers with v2.1 than v2.0 in the transition zone and the peripheral zone [23]. The authors suggested that the PI-RADS v2.1 could improve the interreader agreement and might contribute to improved diagnostic performance compared to v2.0 among radiologists with different levels of expertise.

The radiologists participating in the study highlighted some inconsistencies of PI-RADS v2.1; for example, the PI-RADS assessment of DWI for the transition zone (Score 2). They stated that ‘linear/wedge-shaped hypointense on ADC and/or linear/wedge-shaped hyperintense on high b-value DWI’, whereas linear- or wedge-shaped lesions are typical for the peripheral zone not for the transition zone of the prostate. The radiologists pointed out the subjectivity of radiological assessment in estimating individual parameters and indicated the potential for introducing objective measures that could help them in estimating imaging features.

The interviews we conducted with specialists who had interacted with the SR system allowed us to improve the solution’s usability and confirmed its promise in improving work ergonomics and, by further integration with computational methods, to provide an interface for CAD through structured reporting. 

This study has some limitations. The size of the experimental group is quite small. This study was performed as a pilot study and was not a part of any larger scientific project. We invited to the study a representative group of six radiologists and planned two stages of experiments, especially to observe intrarater dependencies. The founding limitation did not allow for the use of a large number of cases. The median time required to assess a single lesion according to the measured interaction time with the tool during the retrospective study was nine minutes and fifteen seconds; however, this is incomparable to clinical practice, as due to the study design, only single-lesion prostate mpMRIs were evaluated, which is not typical for PCa assessment.

The data generated through the interaction of radiologists with structured reporting systems facilitates research by constructing annotated datasets that can be used to investigate assessment qualities and introduce improvements in diagnostic protocols.

Low interrater agreement in the assessment of mpMRI features negatively affects the quality of the annotations that are assigned to medical examinations. The creation of high-quality reference datasets that could be used for research and the further development of the computational method is crucial to achieving advances in AI-enhanced SR systems. This would require the conductance of a multicenter retrospective study involving multiple experienced radiologists who evaluate representative datasets of prostate imaging. A ‘gold standard’ could then be established by estimating the confidence levels for variables based on expert evaluations. The assessments of multiple experienced radiologists are crucial in capturing the intuitions involved in estimating the values of the defined CDEs;for example, the meaning of moderate signal intensity on T2W images. Establishing the requirements to classify signal intensity as moderate would require multiple ratings of varying imaging characteristics. This expands to other features, particularly those with high interrater variability.

## 5. Conclusions

Our research has demonstrated the need for further work to clarify the concepts and features considered in the PI-RADS assessment. Radiologists differ in their assessment of lesions’ qualities, number, and the probability of their clinical significance. The results show that high-level features that require expert knowledge and subjective interpretation demonstrate decreased agreement between raters. We have demonstrated that it is possible to develop structured reporting systems of radiological assessment in PCa diagnosis that integrate with formal descriptions, which could improve the quality and the reproducibility of data available to refine artificial intelligence algorithms.

## Figures and Tables

**Table 1:** Configuration of defined PI-RADS v2.1 CDEs along with the identified related RadLex terms.

Variable	Label	Related Radlex Terms	Possible Values
lesion_dim_max	Lesion max dimension (mm)	Diameter [RID13432]	<5, ≥5, ≥15
lesion_location	Zone	Zone of prostate [RID38890]	PZ, TZ, Not Available
t2w_present_and_adequate	T2W present and adequate	Adequate [RID39308]	YES, NO
t2w_abnormality	T2W lesion present	Lesion [RID 38780]	YES, NO
t2w_invasive	T2W Invasive	Invasive [RID5680]	YES, NO
t2w_signal_intensity_type	T2W signal intensity type	Signal characteristic [RID6049]	Hypointense, Isointense, Hyperintense
t2w_signal_intensity	T2W signal intensity scale	Signal characteristic [RID6049]	Mild, Moderate, Marked
t2w_uniformity	T2W lesion uniformity	Uniformity descriptor [RID43293]	Homogeneous, Heterogeneous
t2w_focality	T2W focality	Focal [RID5702]	YES, NO
t2w_shape	T2W shape	Morphologic descriptor [RID5863]	Linear, Wedge, Lenticular, Water-Drop
t2w_shape_category	T2W shape category	Morphologic descriptor [RID5863]	Linear, Round, Irregular
t2w_margin	T2W margin	Margin [RID5972]	Indistinct, Obscured, Spiculated, Erased charcoal sign, Partly_Encapsulated, Encapsulated, Well_Defined
t2w_margin_category	T2W margin category	Margin [RID5972]	Circumscribed, Non_Circumscribed
adc_present_and_adequate	ADC present and adequate	Adequate [RID39308]	YES, NO
adc_abnormality	ADC lesion present	Lesion [RID38780]	YES, NO
adc_invasive	ADC invasive	Invasive [RID5680]	YES, NO
adc_signal_intensity_type	ADC signal intensity type	Signal characteristic [RID6049]	Hypointensitivity, Isointensitivity, Hyperintensitivity
adc_signal_intensity	ADC signal intensity scale	Signal characteristic [RID6049]	Mild, Moderate, Marked
adc_focality	ADC focality	Focal [RID5702]	YES, NO
adc_shape	ADC shape	Morphologic descriptor [RID5863]	Linear, Wedge, Lenticular, Water-Drop
adc_shape_category	ADC shape category	Morphologic descriptor [RID5863]	Linear, Round, Irregular
dwi_present_and_adequate	DWI present and adequate	Adequate [RID39308]	YES, NO
dwi_abnormality	DWI lesion present	Lesion [RID38780]	YES, NO
dwi_invasive	DWI invasive	Invasive [RID5680]	YES, NO
dwi_signal_intensity_type	DWI signal intensity type	Signal characteristic [RID6049]	Hypointense, Isointense, Hyperintense
dwi_signal_intensity	DWI signal intensity scale	Signal characteristic [RID6049]	Mild, Moderate, Marked
dwi_focality	DWI focality	Focal [RID5702]	YES, NO
dwi_shape	DWI shape	Morphologic descriptor [RID5863]	Linear, Wedge, Lenticular, Water-Drop
dwi_shape_category	DWI shape category	Morphologic descriptor [RID5863]	Linear, Round, Irregular
dce_present_and_adequate	Is DCE present and adequate?	Adequate [RID39308]	YES, NO
dce_abnormality	Does an abnormality appear on the DCE image?	Lesion [RID38780]	YES, NO
dce_enhancement	Enhancement pattern	Enhancement pattern [RID6058]	Positive_DCE, Negative_DCE
dce_corresponds_to	Corresponds to finding	MR tissue contrast attribute (Mr procedure attribute) [ RID10791]	T2, DWI, Not_Available
dce_bph_features	BPH features on T2	Benign prostatic hyperplasia [RID3784]	YES, NO

**Table 2:** Interobserver agreement of PI-RADS v2.1 CDEs.

		Session 1			Session 2			Overall		
		PA	AC1 (95% CI)	*p*-Value	PA	AC1 (95% CI)	*p*-Value	PA	AC1 (95% CI)	*p*-Value
Modality	Feature									
OVERALL	Lesion >= 1.5 cm	72.3	0.45 (0.26; 0.63)	<0.001	69.2	0.40 (0.21; 0.58)	<0.001	70.7	0.42 (0.29; 0.55)	<0.001
	Zone (selected)	76.9	0.66 (0.51; 0.81)	<0.001	79.4	0.70 (0.56; 0.84)	<0.001	78.1	0.68 (0.58; 0.78)	<0.001
T2W	Abnormality	94.0	0.94 (0.87; 1.00)	<0.001	99.0	0.99 (0.97; 1.00)	<0.001	96.5	0.96 (0.93; 0.99)	<0.001
	Focality	66.6	0.48 (0.30; 0.65)	<0.001	74.4	0.65 (0.49; 0.80)	<0.001	70.5	0.57 (0.45; 0.68)	<0.001
	Homogeneity	63.9	0.38 (0.19; 0.56)	<0.001	62.7	0.37 (0.19; 0.54)	<0.001	63.3	0.37 (0.25; 0.50)	<0.001
	Invasiveness	68.1	0.45 (0.23; 0.66)	<0.001	72.7	0.57 (0.38; 0.77)	<0.001	70.4	0.51 (0.37; 0.65)	<0.001
	Margin	26.5	0.13 (0.06; 0.20)	<0.001	28.2	0.18 (0.11; 0.24)	<0.001	27.3	0.16 (0.12; 0.21)	<0.001
	Margin cat.	73.0	0.56 (0.36; 0.76)	<0.001	69.2	0.50 (0.29; 0.71)	<0.001	71.1	0.53 (0.39; 0.67)	<0.001
	Shape	27.4	0.18 (0.13; 0.23)	<0.001	23.0	0.13 (0.07; 0.18)	<0.001	25.2	0.15 (0.12; 0.19)	<0.001
	Shape cat.	46.2	0.22 (0.10; 0.35)	<0.01	50.6	0.31 (0.16; 0.45)	<0.001	48.4	0.26 (0.17; 0.35)	<0.001
	Signal int.	45.2	0.24 (0.15; 0.33)	<0.001	54.4	0.38 (0.24; 0.52)	<0.001	49.8	0.37 (0.30; 0.44)	<0.001
	Signal int. type	95.3	0.95 (0.90; 1.00)	<0.001	100.0			97.7	0.98 (0.95; 1.00)	<0.001
DWI	Abnormality	85.0	0.81 (0.69; 0.93)	<0.001	94.0	0.93 (0.87; 1.00)	<0.001	89.5	0.87 (0.81; 0.94)	<0.001
	Focality	73.0	0.61 (0.41; 0.80)	<0.001	77.2	0.68 (0.50; 0.86)	<0.001	75.1	0.64 (0.52; 0.77)	<0.001
	Homogeneity	70.0	0.54 (0.33; 0.75)	<0.001	77.0	0.69 (0.53; 0.85)	<0.001	73.6	0.62 (0.49; 0.75)	<0.001
	Invasiveness	65.1	0.42 (0.22; 0.62)	<0.001	70.0	0.52 (0.32; 0.72)	<0.001	67.6	0.47 (0.33; 0.61)	<0.001
	Shape	25.2	0.16 (0.08; 0.23)	<0.001	20.3	0.10 (0.04; 0.15)	<0.01	22.7	0.12 (0.08; 0.17)	<0.001
	Shape cat.	46.8	0.24 (0.10; 0.38)	<0.01	51.2	0.31 (0.17; 0.46)	<0.001	49.0	0.28 (0.18; 0.37)	<0.001
	Signal int.	50.2	0.26 (0.13; 0.40)	<0.001	55.5	0.34 (0.18; 0.50)	<0.001	52.9	0.38 (0.29; 0.47)	<0.001
	Signal int. type	89.0	0.88 (0.75; 1.00)	<0.001	94.6	0.94 (0.87; 1.00)	<0.001	91.9	0.91 (0.84; 0.98)	<0.001
ADC	Abnormality	92.3	0.91 (0.84; 0.99)	<0.001	94.4	0.94 (0.88; 1.00)	<0.001	93.3	0.93 (0.88; 0.98)	<0.001
	Focality	74.2	0.64 (0.46; 0.82)	<0.001	76.4	0.67 (0.52; 0.83)	<0.001	75.3	0.66 (0.54; 0.78)	<0.001
	Homogeneity	65.3	0.49 (0.33; 0.65)	<0.001	73.6	0.63 (0.46; 0.80)	<0.001	69.5	0.56 (0.44; 0.68)	<0.001
	Invasiveness	67.1	0.45 (0.25; 0.65)	<0.001	71.2	0.54 (0.34; 0.75)	<0.001	69.2	0.50 (0.36; 0.64)	<0.001
	Shape	23.5	0.13 (0.08; 0.19)	<0.001	21.1	0.11 (0.05; 0.16)	<0.001	22.3	0.12 (0.08; 0.16)	<0.001
	Shape cat.	46.5	0.23 (0.09; 0.37)	<0.01	50.5	0.30 (0.17; 0.44)	<0.001	48.5	0.27 (0.17; 0.36)	<0.001
	Signal int.	47.4	0.24 (0.08; 0.41)	<0.01	59.9	0.44 (0.27; 0.61)	<0.001	53.6	0.39 (0.29; 0.48)	<0.001
	Signal int. type	94.8	0.95 (0.87; 1.00)	<0.001	100.0			97.4	0.97 (0.94; 1.00)	<0.001
DCE	Abnormality	77.9	0.64 (0.47; 0.82)	<0.001	75.0	0.65 (0.52; 0.78)	<0.001	76.5	0.65 (0.54; 0.75)	<0.001
	BPH features	82.7	0.77 (0.57; 0.95)	<0.001	85.7	0.81 (0.67; 0.95)	<0.001	84.3	0.79 (0.68; 0.91)	<0.001
	Enhancement	79.3	0.75 (0.57; 0.93)	<0.001	75.7	0.60 (0.41; 0.79)	<0.001	77.3	0.68 (0.55; 0.80)	<0.001
	Focality	43.7	0.32 (0.17; 0.47)	<0.001	45.6	0.35 (0.19; 0.51)	<0.001	44.7	0.34 (0.23; 0.44)	<0.001

**Table 3:** Interobserver agreement of the PI-RADS v2.1 category assessments.

	Session 1		Session 2		Overall	
Feature	PA	AC1 (95% CI)	PA	AC1 (95% CI)	PA	AC1 (95% CI)
T2W PI-RADS	47.1	0.35 (0.26; 0.45)	43.8	0.31 (0.19; 0.43)	45.4	0.33 (0.26; 0.41)
DWI PI-RADS	50.0	0.39 (0.28; 0.50)	49.2	0.38 (0.29; 0.47)	49.6	0.39 (0.32; 0.46)
DCE PI-RADS	69.5	0.48 (0.28; 0.68)	69.8	0.42 (0.24; 0.60)	69.6	0.45 (0.31; 0.58)
OVERALL PI-RADS	47.1	0.35 (0.25; 0.46)	42.7	0.30 (0.19; 0.41)	44.9	0.33 (0.26; 0.40)

**Table 4:** Intraobserver agreement of the manual PI-RADS v2.1 assessment.

	Experienced		Inexperienced		Overall	
Feature	PA	AC1 (95% CI)	PA	AC1 (95% CI)	PA	AC1 (95% CI)
T2W PI-RADS	72.3	0.66 (0.55; 0.78)	60.4	0.51 (0.19; 0.43)	66.3	0.59 (0.50; 0.41)
DWI PI-RADS	61.3	0.53 (0.40; 0.66)	61.7	0.53 (0.29; 0.47)	61.5	0.53 (0.44; 0.46)
DCE PI-RADS	75.0	0.51 (0.29; 0.73)	76.8	0.59 (0.24; 0.60)	76.0	0.55 (0.41; 0.69)
OVERALL PI-RADS	67.0	0.60 (0.48; 0.72)	61.5	0.53 (0.19; 0.41)	64.2	0.56 (0.48; 0.65)

## Data Availability

The dataset used and analyzed during the current study is a sub-set of the publicly available database of mpMRI data for prostate lesion classification, which was originally created for the PROSTATEx Challenge (SPIE-AAPM-NCI Prostate MR Classification Challenge) held in conjunction with the 2017 SPIE Medical Imaging Symposium. The PROSTATEx dataset is publicly available through the Cancer Imaging Archive PROSTATEx webpage (https://wiki.cancerimagingarchive.net/pages/viewpage.action?pageId=23691656, accessed on 10 June 2022).
